# Association of *Nicotinamide Phosphoribosyltransferase* (*NAMPT*) Gene Polymorphisms and of Serum NAMPT Levels with Dilated Cardiomyopathy in a Chinese Population

**DOI:** 10.3390/ijms160922299

**Published:** 2015-09-15

**Authors:** Qingyu Dou, Ying Peng, Bin Zhou, Kui Zhang, Jing Lin, Xiaohui Dai, Lin Zhang, Li Rao

**Affiliations:** 1 Department of Cardiology, West China Hospital of Sichuan University, Chengdu 610041, China; E-Mails: ddqqking@126.com (Q.D.); joeypengying2013@163.com (Y.P.); linyun1020@sina.com (J.L.); daixiaohui20061008@126.com (X.D.); 2 Key Laboratory of Obstetric & Gynecologic and Pediatric Diseases and Birth Defects of Ministry of Education, West China Second University Hospital, Sichuan University, Chengdu 610041, China; E-Mails: zb630@163.com (B.Z.); zhanglin@scu.edu.cn (L.Z.); 3 Laboratory of Molecular Translational Medicine, West China Institute of Women and Children’s Health, West China Second University Hospital, Sichuan University, Chengdu 610041, China; 4 Department of Forensic Pathology, Sichuan University, Chengdu 610041, China; E-Mail: zhk@scu.edu.cn

**Keywords:** DCM, NAMPT, sirtuin-1(SIRT1), SNP, survival analysis

## Abstract

Nicotinamide phosphoribosyltransferase (NAMPT) has crucial roles for myocardial development, cardiomyocyte energy metabolism and cell death/survival by regulating NAD^+^-dependent sirtuin-1 (SIRT1) deacetylase. This study aimed to determine if the single nucleotide polymorphisms (SNPs) of the *NAMPT* gene may affect the susceptibility and prognosis for patients with dilated cardiomyopathy (DCM) and to describe the association of serum NAMPT levels with clinical features of DCM. Three SNPs (rs61330082, rs2505568, and rs9034) were analyzed by the polymerase chain reaction-restriction fragment length polymorphism method in a case-control study of 394 DCM patients and 395 controls from China. Serum NAMPT levels were measured by enzyme-linked immunosorbent assay kits. The homozygote for the minor allele at rs2505568 and rs9034 could not be detected in this study. Rs9034 *T* allele and *CT* genotype were associated with increased DCM risk (OR: 1.63, 95% CI = 1.16–2.27, *p* = 0.005 and OR: 1.72, 95% CI = 1.20–2.50, *p* = 0.0027, respectively). Nominally significant decreased DCM risk was found to be associated with the *A* allele and *AT* genotype of rs2505568 (OR: 0.48, 95% CI = 0.35–0.67, *p* < 0.0001 and OR: 0.44, 95% CI = 0.31–0.62, *p* < 0.0001, respectively), but it should be interpreted with caution because of Hardy-Weinberg disequilibrium in the control group. Of five haplotypes constructed, *TAC* (rs61330082-rs2505568-rs9034) was a protective haplotype to DCM (OR: 0.22, 95% CI = 0.13–0.39, *p* = 1.84 × 10^−8^). The Cox multivariate survival analysis indicated that the rs9034 *CT* genotype (hazard ratio (HR): 0.59, 95% CI = 0.37–0.96, *p* = 0.03) was an independently multivariate predictor for longer overall survival in DCM patients. Serum NAMPT levels were significantly higher in the DCM group than controls (*p* < 0.0001) and gradually increased with the increase of New York Heart Association grade in DCM patients. However, there was a lack of association of the three SNPs with serum NAMPT levels. Spearman correlation test revealed that the NAMPT level was positively associated with brain natriuretic peptide (*r* = 0.56, *p* = 0.001), left ventricular end-diastolic diameter (*r* = 0.293, *p* = 0.011) and left ventricular end-diastolic volume (*r* = 0.294, *p* = 0.011). Our study suggested that NAMPT may play an important role in the development of DCM.

## 1. Introduction

Dilated cardiomyopathy (DCM) is a heart muscle disorder characterized by dilatation and systolic impairment of the left or both ventricles in the absence of hypertension, coronary artery disease or valvular abnormalities [1]. DCM is the most frequent cause of heart failure (HF) in the young [2] and the most common indication for cardiac transplantation [3]. The etiology of the disease has not yet been fully unraveled, involving both genetic and environmental factors. To date, mutations in more than 50 genes have been implicated in the development of DCM. Genes encoding for sarcomeric, cytoskeletal, nuclear membrane, dystrophin-associated glycoprotein complex and desmosomal proteins are the principal genes involved [4]. However, these mutations explain only a minority of the etiology of DCM. Most DCMs are sporadic and nonfamilial with multifactorial causes linked to genetic susceptibility. Many genetic polymorphisms have been shown to be associated with an increased risk of developing DCM [5,6]. Therefore, genetic studies should not be restricted to familial DCM.

Nicotinamide phosphoribosyltransferase (NAMPT), also named pre-B cell enhancing factor (PBEF) or visfatin, is a key enzyme for synthesizing nicotinamide adenine dinucleotide (NAD) and exists in two known forms, intracellular NAMPT (iNAMPT), and a secreted form, extracellular NAMPT (eNAMPT) [7,8]. iNAMPT participates in the salvage pathway of NAD synthesis and NAD plays a vital role in energy metabolism, serving as a cofactor of histone deacetylase sirtuins [9]. Among them, sirtuin-1(SIRT1) is crucially involved in regulation of myocardial development, cardiomyocyte energy metabolism, production of reactive oxygen species and signaling in regards to cell death/survival [10]. On the other hand, eNAMPT, mostly in the form of serum NAMPT, likely functions as a potential inflammatory cytokine in diverse biological contexts, including acute lung injury, cancer, rheumatoid arthritis, atherosclerosis, and heart failure [11,12]. NAMPT has been shown to react with a large number of inflammatory cytokines, such as IL-6 and TNFα [13]. These cytokines are known to mediate pro-inflammatory and detrimental effects in the progression of cardiomyopathy and heart failure [14].

A study has shown that serum NAMPT level is a biomarker and independent risk factor of systolic heart failure [15]. Systolic heart failure characterized by left ventricular dilation and systolic dysfunction is the main manifestation of DCM. Some other cardiomyopathies, such as hypertrophic cardiomyopathy and restrictive cardiomyopathy, cause diastolic heart failure with nondilated ventricles and normal systolic function. Therefore, the *NAMPT* gene may be involved in the pathogenesis of DCM. Meanwhile, polymorphisms in *NAMPT* with susceptibility and prognosis of DCM have not been investigated. We conducted this case-control study to clarify the hypothesis that the SNPs of the *NAMPT* gene may affect the susceptibility and prognosis for patients with DCM and to describe the association of serum NAMPT levels with clinical features of DCM.

## 2. Results

### 2.1. Baseline Characteristics of Controls and DCM Patients

Table 1 summarizes the baseline clinical characteristics of the patients with DCM and control groups. As shown in Table 1, age and gender distribution did not differ between DCM patients and controls (*p* > 0.05). Compared to controls, DCM patients had higher heart rate, creatinine, brain natriuretic peptide (BNP), left ventricular end-diastolic diameter (LVEDD), and lower left ventricular ejection fraction (LVEF), systolic blood pressure (SBP), diastolic blood pressure (DBP) (*p* < 0.05) and more severe NYHA functional class. All DCM patients were treated according to the guidelines for medical treatment of heart failure.

**Table 1:** Baseline characteristics of controls and dilated cardiomyopathy (DCM) patients.

Variable	Control *n* = 395	DCM *n* = 394
Gender (male/female)	261/134	259/135
Age (years)	45.32 ± 11.67	47.01 ± 14.52
SBP (mmHg)	126.92 ± 15.32	112.83 ± 17.49 *
DBP (mmHg)	74.43 ± 12.45	59.78 ± 10.29 *
Heart rate, beats/min	78.56 ± 19.21	85.72 ± 14.45 *
NYHA	I: 321; II: 74	II: 68; III: 273; IV: 53
Creatinine (μmol/L)	76.35 ± 24.67	89.68 ± 28.47 *
BNP (pg/mL)	97.45 ± 32.96	7253.02 ± 5431.61 *
LVEF (%)	66.11 ± 8.07	33.13 ± 13.24 *
LVEDD (mm)	47.61 ± 5.27	66.90 ± 11.04 *
Diuretics, *n* (%)	0	374 (94.9%)
ACEI/ARB, *n* (%)	0	323 (82.0%)
Beta-blockers, *n* (%)	0	282 (71.6%)
Digoxin, *n* (%)	0	295 (74.9%)
Spironolactone, *n* (%)	0	357 (90.6%)

Data are presented as the mean ± SD or number (%); NYHA, New York Heart Association; SBP, systolic blood pressure; DBP, diastolic blood pressure; BNP, brain natriuretic peptide; LVEF, left ventricular ejection fraction; LVEDD, left ventricular end-diastolic diameter; ACEI, angiotensin-converting enzyme inhibitor; ARB, angiotensin receptor blocker; DCM: dilated cardiomyopathy; * Control *vs*. DCM *p* < 0.05.

### 2.2. Distribution of Genotype and Allele Frequencies between DCM Patients and Controls

Three SNPs of *NAMPT* gene including rs61330082 (T>C), rs2505568 (T>A) and rs9034 (C>T) were selected for analyses. The rs61330082 in the promoter region was selected from the online prediction website (Available online: http://www-bimas.cit.nih.gov/molbio/proscan/), which may potentially affect the promoter region. Both rs2505568 and rs9034 exist in the 3′ untranslated region. They were selected from the online prediction website (Available online: http://www.mirbase.org/index.shtml) [16], and these two SNPs were potential binding sites for miRNAs.

All three SNPs of the *NAMPT* gene were successfully genotyped in 394 patients with DCM and 395 control subjects. Using the Hardy-Weinberg equation to check the genetic distribution within the two subject groups, we noted that rs9034 genotypes in DCM group and rs2505568 genotypes in control group did not conform to the Hardy-Weinberg equilibrium (*p* = 0.0079 and *p* = 0.00071, respectively). The other observed genotyped frequencies in both DCM patients and controls were consistent with the Hardy-Weinberg equilibrium (*p* > 0.05). Genotype distributions and allele frequencies in patients and controls are shown in Table 2. The absence of homozygote for the minor allele at rs2505568 and rs9034 was consistent with the previous data of a Chinese population [17]. Due to the absence of homozygote for the minor allele, only dominant genetic model analysis was available for both rs2505568 and rs9034. After Bonferroni correction for multiple testing, rs9034 *T* allele and *CT* genotype were associated with increased DCM risk (OR: 1.63, 95% CI = 1.16–2.27, *p* = 0.005 and OR: 1.72, 95% CI = 1.20–2.50, *p* = 0.0027 in the dominant genetic model, respectively). Nominally significant decreased DCM risk was also found to be associated with the *A* allele and *AT* genotype of rs2505568 (OR: 0.48, 95% CI = 0.35–0.67, *p* < 0.0001 and OR: 0.44, 95% CI = 0.31–0.62, *p* < 0.0001 in the dominant genetic model, respectively). For SNP rs61330082 polymorphism, there were no differences of the allele and genotype frequencies between DCM and control groups. We also obtained the statistical power of 0.81 and 0.99 for the two significant SNPs identified, rs9034 and rs2505568, respectively. This shows that our sample size of 789 was adequate and the study was sufficiently equipped to detect the true association of these two SNPs with DCM. However, the association of rs2505568 polymorphism with DCM should be interpreted more cautiously because of Hardy-Weinberg disequilibrium in the control group.

**Table 2:** Distribution of SNP in *NAMPT* among cases and controls and their association with DCM risk.

Model		Cases (*n* = 394)	Controls (*n* = 395)	OR (95% CI)	*p* Value
		*n* (%)	*n* (%)		
**rs61330082**					
	Genotype				
Codominant	*TT*	93 (23.6%)	103 (26.1%)	1.00	
	*CT*	197 (50.0%)	201 (50.9%)	1.09 (0.77–1.53)	0.49
	*CC*	104 (26.4%)	91 (23.0%)	1.27 (0.83–1.93)	
Dominant	*TT*	93 (23.6%)	103 (26.1%)	1.00	0.42
	*CT/CC*	301 (76.4%)	292 (73.9%)	1.14 (0.83–1.58)	
Recessive	*TT/CT*	290 (73.6%)	304 (77.0%)	1.00	0.27
	*CC*	104 (26.4%)	91 (23.0%)	1.20 (0.87–1.67)	
Overdominant	*TT/CC*	197 (50.0%)	194 (49.1%)	1.00	0.80
	*CT*	197 (50.0%)	201 (50.9%)	0.97 (0.73–1.28)	
	Allele				
	*T*	383 (48.6%)	407 (51.5%)	1.00	0.25
	*C*	405 (51.4%)	383 (48.5%)	1.12 (0.92–1.34)	
**rs2505568**					
	Genotype				
Dominant	*TT*	334 (84.8%)	280 (70.9%)	1.00	***<0.0001***
	*AT*	60 (15.2%)	115 (29.1%)	***0.44 (0.31–0.62)***	
	Allele				
	*T*	728 (92.4%)	675 (85.4%)	1.00	***<0.0001***
	*A*	60 (7.6%)	115 (14.6%)	***0.48 (0.35–0.67)***	
**rs9034**					
	Genotype				
Dominant	*CC*	301 (76.4%)	335 (84.8%)	1.00	***0.0027***
	*CT*	93 (23.6%)	60 (15.2%)	***1.72 (1.20–2.50)***	
	Allele				
	C	695 (88.2%)	730 (92.4%)	1.00	***0.005***
	T	93 (11.8%)	60 (7.6%)	***1.63 (1.16–2.27)***	

Significant *p* values after multiple testing adjustment (*p* < 0.017) are shown in italic bold; SNP, single nucleotide polymorphism; DCM, dilated cardiomyopathy; OR, odd ratio; CI, confidence interval.

### 2.3. Linkage Disequilibrium and Haplotype Analysis

We estimated linkage disequilibrium (LD) among the three variants by using SHESIS software (Available online: http://analysis.bio-x.cn/myAnalysis.php). The SNP rs61330082, rs2505568, and rs9034 were not in linkage disequilibrium with each other (*r*^2^ = 0.000, *r*^2^ = 0.047 and *r*^2^ = 0.006, respectively). In other words, the three SNPs were almost independent of each other. Therefore, every SNP in our study has different significance and cannot be replaced by each other. We also found *TAC* (rs61330082-rs2505568-rs9034) haplotype which included two low-risk alleles (*A* allele at rs2505568 and *C* allele at rs9034) was a protective haplotype to DCM (OR: 0.22, 95% CI = 0.13–0.39, *p* = 1.84 × 10^−8^) after multiple testing adjustment (Table 3).

**Table 3:** Haplotype frequencies of *NAMPT* gene in the patients with DCM and in controls.

Haplotype	SNP Positions			Cases *n* (%)	Controls *n* (%)	OR (95% CI)	*p* Value
	rs61330082	rs2505568	rs9034				
*a*	*C*	*A*	*C*	38 (4.9%)	48 (6.0%)	0.82 (0.53–1.27)	0.36
	*C*	*A*	*T*	2 (0.3%)	1 (0.1%)	-	-
*b*	*C*	*T*	*C*	297 (37.7%)	279 (35.3%)	1.16 (0.94–1.42)	0.17
*c*	*C*	*T*	*T*	67 (8.6%)	56 (7.0%)	1.28 (0.88–1.85)	0.19
*d*	*T*	*A*	*C*	15 (1.9%)	65 (8.3%)	***0.22(0.13–0.39)***	***1.84×10^−8^***
	*T*	*A*	*T*	4 (0.5%)	1 (0.2%)	-	-
*e*	*T*	*T*	*C*	335 (43.8%)	338 (42.8%)	1.09 (0.90–1.34)	0.36
	*T*	*T*	*T*	19 (2.4%)	3 (0.3%)	-	-

Haplotypes with frequency less than 3.0% were not analyzed; significant *p* value after multiple testing adjustment (*p* < 0.01) is shown in italic bold; OR, odd ratio; CI, confidence interval.

### 2.4. Cox Regression Analysis of Cardiac Death in Patients with DCM

**Figure 1:**
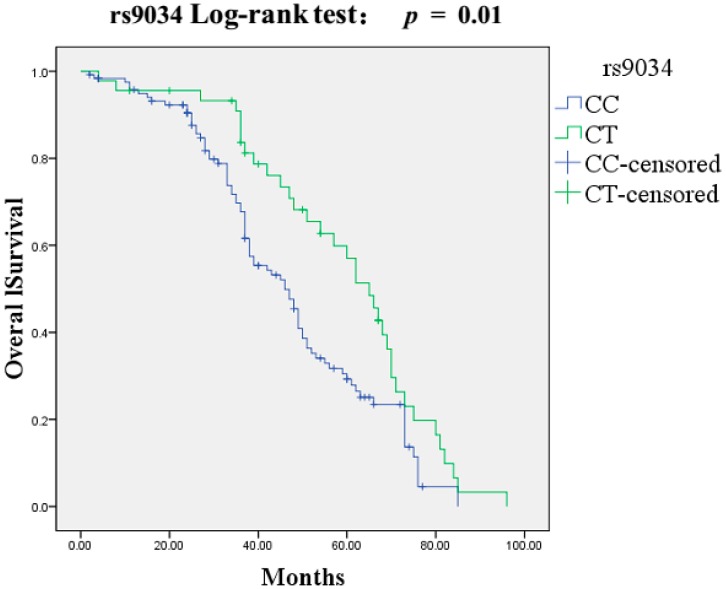
Kaplan-Meier survival curves free of cardiac death for 175 DCM patients based on rs9034.

In survival analysis with the three SNPs of *NAMPT* gene, 175 DCM patients were followed for a mean period of 71.3 ± 32.4 months. During follow up, all patients received continuous medication treatment and no one underwent heart transplantation. One hundred and eighteen patients (67.4%) died, 86 of them due to pump failure and 32 due to cardiac sudden death. The univariate analysis demonstrated that the rs9034 *CT* genotype presented longer overall survival than *CC* genotype (HR: 0.59, 95% CI = 0.39–0.89, *p* = 0.01) (Figure 1). Additionally, female gender (HR: 0.66, 95% CI = 0.45–0.98, *p* = 0.04), increased LVEF (HR: 0.97, 95% CI = 0.59–0.99, *p* = 0.001) and use of beta-blocker therapy (HR: 0.79, 95% CI = 0.66–0.95, *p* = 0.01) were significant predictors for survival in patients with DCM, while advanced NYHA class (HR: 1.39, 95% CI = 1.03–1.87, *p* = 0.03) and BNP > 7897 pg/mL (HR: 1.27, 95% CI = 1.06–1.52, *p* = 0.01) were associated with cardiac death in DCM patients. No statistically significant association for rs61330082 and rs2505568 polymorphisms with overall survival time was found in univariate survival analysis. Since *TAC* (rs61330082-rs2505568-rs9034) was a protective haplotype to DCM, we also evaluated the association between haplotypes with cardiac death. However, no haplotype was associated with cardiac death according to our analysis. This negative result may be influenced by our small sample size (175 patients) and very low proportion of some haplotypes. For instance, the *TAC* (rs61330082-rs2505568-rs9034) haplotype only comprised 1.9% of all haplotypes in cases. The variables including SNP rs9034, age, gender, creatinine, NYHA functional class, LVEF, BNP > 7897 pg/mL and beta-blocker therapy were analyzed in the subsequent multivariate Cox model. The Cox multivariate analysis indicated that the SNP rs9034 *CT* genotype (HR: 0.59, 95% CI = 0.37–0.96, *p* = 0.03) together with increased LVEF (HR: 0.98, 95% CI = 0.95–1.00, *p* = 0.04) were independently multivariate predictors for longer overall survival in DCM patients (Table 4).

**Table 4:** Cox regression analysis of cardiac death in patients with DCM.

Characteristics		Overall Survival					
		Univariate Survival Analysis			Multivariate Survival Analysis		
		HR	95% CI	*p* Value	HR	95% CI	*p* Value
**Genotype**							
**rs61330082**							
Dominant	*TT*	1					
	*CT/CC*	1.09	0.87–1.37	0.45	-	-	-
Recessive	*CT/TT*	1					
	*CC*	1.11	0.92–1.35	0.27	-	-	-
Overdominant	*CC/TT*	1					
	*CT*	1.06	0.74–1.54	0.72	-	-	-
**rs2505568**							
Dominant	*TT*	1					
	*AT*	0.91	0.56–1.47	0.69	-	-	-
**rs9034**							
Dominant	*CC*	1					
	*CT*	***0.59***	***0.39–0.89***	***0.01***	***0.59***	***0.37–0.96***	***0.03***
**Haplotype**							
a = *CAC*	*−− ^#^*	1					
	*−a/aa*	1.64	0.88–3.03	0.11	-	-	-
b = *CTC*	*−−*	1					
	*−b/bb*	1.17	0.79–1.72	0.42	-	-	-
c = *CTT*	*−−*	1					
	*−c/cc*	0.89	0.70–1.13	0.33	-	-	-
*d = TAC*	*−−*	1					
	*−d/dd*	0.90	0.61–1.32	0.59	-	-	-
*e = TTC*	*−−*	1					
	*−e/ee*	0.98	0.88–1.10	0.75	-	-	-
Age		1.00	0.99–1.02	0.74	1.00	0.98–1.02	0.94
Gender (female)		***0.66***	***0.45*–*0.98***	***0.04***	0.70	0.44–1.13	0.14
NYHA		***1.39***	***1.03*–*1.87***	***0.03***	0.95	0.64-1.40	0.78
LVEDD (mm)		1.00	0.98–1.02	0.92	-	-	-
LVEF (%)		***0.97***	***0.95*–*0.99***	***0.001***	***0.98***	***0.95*–*1.00***	***0.04***
Creatinine (μmol/L)		1.01	1.00-1.03	0.08	1.01	0.99–1.01	0.19
BNP * >7897 pg/mL		***1.27***	***1.06*–*1.52***	***0.01***	1.21	0.98–1.51	0.08
Diuretics		1.05	0.86–1.29	0.61	-	-	-
ACEI/ARB		0.95	0.66–1.36	0.77	-	-	-
Digoxin		0.96	0.64–1.44	0.86	-	-	-
Spironolactone		0.89	0.73–1.09	0.26	-	-	-
Beta-blocker		***0.79***	***0.66*–*0.95***	***0.01***	0.81	0.63–1.40	0.78

^#^ The minus sign “−” denotes any haplotype. For example: “−a” indicates “a” haplotype in combination with any other haplotype. “aa” indicated the combination of two “a” haplotypes. “−a/aa” indicated individuals carrying “a” haplotype. “*−−*” indicated individuals carrying any other haplotype except for “a” haplotype; BNP * was redefined as categorical according to median BNP plasma level (7897 pg/mL); The variables analyzed in the multivariate Cox model included SNP rs9034, age, gender, creatinine, NYHA functional class, LVEF, BNP > 7897 pg/mL and beta-blocker therapy; *p* < 0.05 was considered to be statistically significant and the values were given in italic bold font; SNP, single nucleotide polymorphism; DCM, dilated cardiomyopathy; HR, hazard ratio; CI, confidence interval; NYHA, New York Heart Association; BNP, brain natriuretic peptide; LVEF, left ventricular ejection fraction; LVEDD, left ventricular end-diastolic diameter; ACEI, angiotensin-converting enzyme inhibitor; ARB, angiotensin receptor blocker.

### 2.5. Association of Serum NAMPT Levels with Clinical Features of DCM

**Figure 2:**
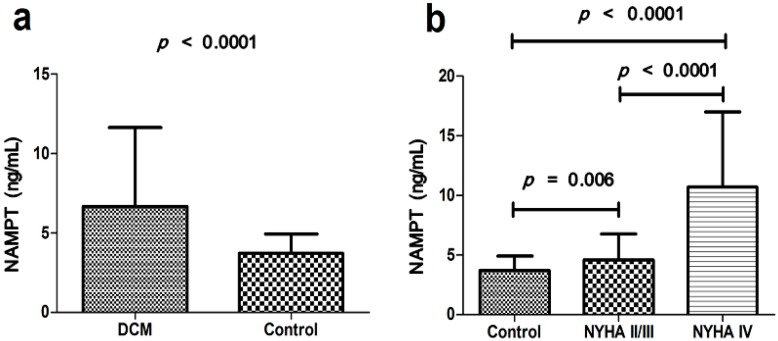
(**a**) Serum NAMPT levels were significantly increased in DCM blood samples (*p* < 0.0001); and (**b**) Serum NAMPT levels significantly increased in NYHA IV class group than NYHA II/III class group and the controls (10.67 ± 6.23, 4.59 ± 2.17 and 3.71 ± 1.21 ng/mL, all *p* < 0.0001). Serum NAMPT levels in NYHA II/III class group significantly increased compared to controls (4.59 ± 2.17 and 3.71 ± 1.21 ng/mL, *p* = 0.006). Data are presented as means ± SD.

**Figure 3:**
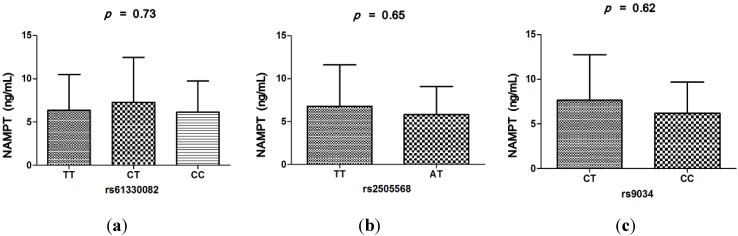
(**a**) No significant relationship was found between serum NAMPT levels and polymorphism of rs61330082 (*p* = 0.73); (**b**) No significant relationship was found between serum NAMPT levels and polymorphism of rs2505568 (*p* = 0.65); and (**c**) No significant relationship was found between serum NAMPT levels and polymorphism of rs9034 (*p* = 0.62). Data are presented as means ± SD.

Serum NAMPT levels were significantly higher in the DCM group compared with controls (6.69 ± 4.97 and 3.71 ± 1.21 ng/mL, *p* < 0.0001) (Figure 2a). There were significant increases of serum NAMPT levels in NYHA IV class group than NYHA II/III class group and the controls (10.67 ± 6.23, 4.59 ± 2.17 and 3.71 ± 1.21 ng/mL, all *p* < 0.0001). The NAMPT levels in NYHA II/III class group were significantly increased compared to controls (4.59 ± 2.17 and 3.71 ± 1.21 ng/mL, *p* = 0.006) (Figure 2b). However, no significant relationship was found between serum NAMPT levels and genotypes of the three SNPs (Figure 3a–c).

**Figure 4:**
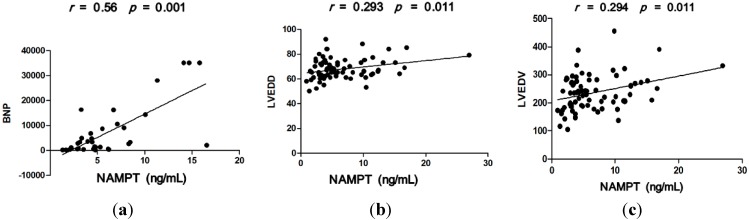
(**a**) Serum NAMPT levels are positively correlated with BNP of DCM patients (*r* = 0.56, *p* = 0.001); (**b**) Serum NAMPT levels are positively correlated with LVEDD of DCM patients (*r* = 0.293, *p* = 0.011); (**c**) Serum NAMPT levels are positively correlated with LVEDV of DCM patients (*r* = 0.294, *p* = 0.011).

A significant positive correlation between serum NAMPT levels and BNP (*r* = 0.56, *p* = 0.001) in DCM patients was found (Figure 4a). Additionally, we also observed a significant correlation between serum NAMPT levels and left ventricular end-diastolic diameter (LVEDD) (*r* = 0.293, *p* = 0.011) (Figure 4b) and left ventricular end-diastolic volume (LVEDV) (*r* = 0.294, *p* = 0.011) (Figure 4c). There were no correlations between serum NAMPT levels and left ventricular stroke volume, ejection fraction and fractional shortening (*r* = 0.10, *p* = 0.39; *r* = −0.107, *p* = 0.362 and *r* = 0.027, *p* = 0.85, respectively).

## 3. Discussion

NAMPT is a critical rate-limiting enzyme of the NAD^+^ salvage pathway for numerous cellular functions including regulation of the SIRT1 [18]. Recent studies have clearly demonstrated the NAMPT-SIRT1 pathway regulates metabolic response, cellular differentiation and life span, cell death, and other important biological events in a number of different cell types, including cardiac myocytes [19]. Alcendor *et al.* reported that moderate overexpression of SIRT1 up to 7.5-fold attenuated age-dependent cardiac dysfunction and oxidative stress-induced apoptosis in mouse hearts, whereas a higher level (12.5-fold) of overexpression of SIRT1 increased apoptosis and hypertrophy and decreased cardiac function [20]. Similarly, Kawashima *et al.* demonstrated that constitutive cardiac-specific overexpression of SIRT1 at a high level (20-fold) caused dilated cardiomyopathy and that moderate (6.8-fold) overexpression of SIRT1 impaired cardiac diastolic function [21]. Oka *et al.* also provided evidence that overexpression of SIRT1 may deteriorate mitochondrial function and exacerbate cardiac dysfunction by suppressing expression of genes regulated by estrogen-related receptors in cardiomyocytes [22]. On the other hand, SIRT1-deficient mice also showed a progressive dilated cardiomyopathy strongly associated with mitochondrial dysfunction, and SIRT1 plays an essential role in the maintenance of mitochondrial integrity and modulates the Mef2 transcription factors in the heart [23]. These results may suggest that the significant role of SIRT1 in the context of cardiac function and the beneficial effect of SIRT1 may be confined to a window of optimal activity. In addition, NAMPT is prominently overexpressed along with SIRT1 in human prostate cancer cells and SIRT1 is a key downstream target of NAMPT for prostate cancer cell growth and survival [18]. Most importantly, Imai recently proposed a new concept “NAD World” with NAMPT as a driver and SIRT1 as a mediator [24] and the regulation of SIRT1 is found to be dependent on available NAD and hence on NAMPT activity [19,25]. Hence, dysregulated NAMPT, and by regulating potentially SIRT1, may be implicated in pathophysiologic mechanisms of DCM.

Recent evidence indicate that NAMPT may also have non-enzymatic functions as an inflammatory cytokine since its serum levels are increased in various inflammatory disorders [12], including sepsis [26], acute lung injury [27], rheumatoid arthritis (RA) [28], inflammatory bowel disease (IBD) [29], psoriasis [30] and myocardial infarction (MI) [31]. The increased levels of NAMPT in serum correlated with myocardial enzymes in patients with acute ST-elevation myocardial infarction patients [32]. There is also a significantly positive correlation between plasma concentrations of NAMPT and high sensitivity *C*-reactive protein (hs-CRP) and IL-6 in the patients with stable angina pectoris [33]. Our results found that NAMPT levels gradually increased with the increase of NYHA grade in patients with heart failure. In addition, serum NAMPT levels positively correlated with and BNP levels. To our knowledge, this study also revealed a novel link between serum NAMPT levels and LVEDD/LVEDV in DCM patients for the first time. These findings indicate that NAMPT might be associated with pro-inflammatory response. However, in a recent study [34], high NAMPT expression was associated with a favorable cardiac functional status accompanied by suppressed cardiac TNFα and IL-6 expression in DCM patients. The study indicated serum NAMPT suppresses directly or indirectly the expression of important cytokines (IL-6 and TNFα) involved in heart failure progression in DCM patients. So far, serum NAMPT seems to stimulate as well as suppress inflammatory signals in different models depending on its concentration and on the degree of activation of exposed cells. Taken together, serum NAMPT, by regulating the expression of different cytokines such as TNFα and IL-6, takes part in the heart failure progression in DCM patients.

The human *NAMPT* gene maps to a region on chromosome 7q22.2 and consist of 11 exons. The associations between polymorphisms in *NAMPT* gene and diseases have not been extensively investigated. It has been reported that rs61330082 in NAMPT was statistically associated with risk for ARDS [35] and rs2505568 was significantly associated with bladder cancer risk [17]. Rs9034 was statistically associated with recurrence-free death in bladder cancer patients [17]. The NAMPT rs1319501 associates with increased MI risk in young women [36]. No significant association for the NAMPT rs9770242 and rs59744560 polymorphisms with RA risk was found in the USA [37]. In our study, the rs61330082 polymorphism is located in the promoter region. SNPs in gene promoter region could have an impact on gene expression mainly by influencing the binding affinity of transcription factors [38,39]. Both rs2505568 and rs9034 exist in the 3′untranslated region, and these two SNPs are potential binding sites for miRNAs. miRNAs are small noncoding RNAs of ~22 nucleotides that regulate gene expression, primarily by partially complementary binding to the 3′-UTR of target messenger RNA (mRNA); this leads to mRNA cleavage or translation repression [40]. SNPs within miRNA binding sites could alter translation of target mRNA [41,42]. Our study demonstrated that rs9034 of *NAMPT* was associated with susceptibility and prognosis of DCM and *TAC* (rs61330082-rs2505568-rs9034) was a protective haplotype to DCM after Bonferroni correction for multiple testing. Rs2505568 also had nominally significant association with DCM risk, but the genotype distribution in control group was inconsistent with Hardy-Weinberg equilibrium. All the controls from a routine health survey in our study were from the Han population living in Sichuan Province of southwestern China. A selection bias (population, geographical factors, stratification) may have occurred because of inclusion and exclusion criteria of this study. As a matter of fact, approximately 10% of all genotype-phenotype association studies show deviation from Hardy-Weinberg equilibrium [43], and therefore the results of our study cannot be considered “abnormal”. Deviations from Hardy-Weinberg equilibrium in control subjects may cause bias in estimating the allele-based estimates of genetic effects. Therefore, the association of rs2505568 polymorphism with DCM should be interpreted more cautiously.

Interestingly, the rs9034 CT genotype presented longer overall DCM survival, while also correlating with increased DCM risk in our study. Studies performed with cardiac-specific SIRT1 transgenic mouse model showed that SIRT1 exhibits complicated regulatory functions in the context of cardiac function; that is, depending on the magnitude of SIRT1 expression, it can be beneficial or harmful. Overexpression of SIRT1 at a high level (20-fold) or SIRT1-deficiency caused dilated cardiomyopathy [21,23]. A low to moderate expression of SIRT1 (2.5–7.5-fold over endogenous levels) was found to be protective against age dependent increase in cardiac apoptosis and cardiac dysfunction [20]. Therefore, NAMPT-SIRT1 pathway is critical not only for disease induction but also for counter regulatory mechanisms, perhaps causing a significantly divergent outcome in DCM progression. This result indicated the distinct genetic contributions of rs9034 CT genotype in controlling the onset and outcome of DCM. Regrettably, there is no report about the SNP rs9034 and rs2505568 effects on *NAMPT* gene or even the protein until now. The mechanism with which they influenced the risk or prognosis of DCM needs further study.

Our study found there was a lack of association of the three SNPs genotypes with NAMPT serum levels. It is noteworthy that the effects of NAMPT as a cytokine are mediated via its “extracellular” functions as eNAMPT not via its major intracellular end products (*i.e*., NAD) and thereby via its enzyme property [44]. The serum NAMPT concentrations assessed by ELISA in our study referred to secretion of extracellular NAMPT. We do not know the relationships between the three SNPs and intracellular NAMPT levels. The three SNPs may influence more crucial iNAMPT levels which play an important role in regulating energy metabolism by modulating SIRT1 activity. On the other hand, the level of serum NAMPT mediating inflammatory information is not influenced by these three SNPs and should be evaluated separately from its enzyme function, reflecting a different role of NAMPT involved in the pathogenesis of DCM.

## 4. Materials and Methods

### 4.1. Study Subjects

This case-control study was carried out within 394 unrelated DCM patients recruited from the West China Hospital of Sichuan University during the period 2002–2013. The clinical diagnosis of DCM was based on patient’s history, physical examination, electrocardiogram, echocardiography and coronary angiograph, according to the criteria established by the 1995 World Health Organization/International Society and Federation of Cardiology Task Force on the Classification of Cardiomyopathy (before 2006) [45] and the 2006 American Heart Association Scientific Statement on the Classification of Cardiomyopathy (after 2006) [46]. Three hundred and ninety-five healthy unrelated subjects from a routine health survey were enrolled as controls. The control subjects had no evidence of organic cardiac disease and cardiac dysfunction, and their echocardiogram results were normal. All subjects were from the Han population living in Sichuan Province of southwestern China. Patients with a history of hypertension, coronary heart disease, cardiac valve disease, tachyarrhythmia, heavy alcohol intake, acute viral myocarditis, systemic diseases of putative autoimmune origin, or skeletal myopathies were intentionally excluded. The present study was approved by the hospital ethics committee and all subjects gave written informed consent to participate.

### 4.2. Patient Follow-up

A total of 175 patients who left a telephone number were scheduled for follow up every three months. The clinical follow-up was performed in a blind manner with respect to a patient’s genetic status. The end point during follow-up was cardiac death, including death due to pump failure or sudden cardiac death.

In the initial evaluation, all the clinical data was collected according to the medical records. The serum BNP levels were detected by enzyme-linked immunosorbent assay kits in laboratory department of West China Hospital, Sichuan University. The echocardiographic measurements were performed in all patients using Philips Sonos7500 and iE33 echocardiography system (Philips Medical Systems, Bothell, WA, USA) with a S5-1 broadband phased-array transducer (1–5 MHz). A comprehensive 2D and Doppler echocardiography was performed according to the recommendations of the American Society of Echocardiography [47]. Left ventricular dimensions (left ventricular end-diastolic diameter, LVEDD) and (left ventricular end-systolic diameter, LVESD) were measured with M-mode echocardiography by using the left parasternal window. Left ventricular ejection fraction (EF) was determined by apical two-and four-chamber views with the modified Simpson rule [47].

### 4.3. SNP Selection, DNA Isolation and Genotyping

Genomic DNA of each individual was extracted from 200 uL EDTA-anticoagulated peripheral blood samples by a DNA isolation kit from Bioteke (Beijing, China) and the procedure was performed according to the manufacturer’s instructions. Genotyping of *NAMPT* polymorphisms was performed using the polymerase chain reaction (PCR)-restriction fragment length polymorphism (RFLP) method. The primers and restriction enzymes used in the genotyping analysis are listed in Table 5. PCR-RFLP was carried out as follows: DNA fragments containing the polymorphism were amplified in a total volume of 25 μL, including 2.5 μL 10× PCR buffer, 1.5 mmol/L MgCl_2_, 0.15 mmol/L dNTPs, 0.5 μmol/L each primer, 100 ng of genomic DNA, and 1 U of *Taq* DNA polymerase. Both of the PCR conditions were 94 °C for 4 min, followed by 32 cycles of 30 s at 94 °C, 30 s at 62 °C, and 30 s at 72 °C, with a final elongation at 72 °C for 10 min. PCR products were digested with corresponding restriction enzyme for 2 h and analyzed by 6% polyacrylamide gels with silver staining. About 10% of the samples were randomly selected to carry out the repeated assays, and the results were 100% concordant.

### 4.4. Serum NAMPT Determination

Plasma samples from the patients and healthy controls were separated from venous blood at room temperature, and stored at −70 °C until use. The quantity determination of plasma NAMPT levels of 113 DCM patients and 137 controls was performed by enzyme-linked immunosorbent assay (ELISA) kits (Uscn Life Science, Wuhan, China) following the manufacturer’s protocol. Developed color reaction as measured as OD450 units on an ELISA reader (RT-6000, Beijing, China). The concentration of plasma NAMPT was determined by using standard curve constructed with the kit’s standards over the range of 15.6–1000 pg/mL.

**Table 5:** Polymorphic SNPs markers, PCR primers, restriction enzymes and corresponding alleles.

Marker	Primer Sequence	Major/Minor Gene	Product (bp)	Annealing Temperature (°C)	Restriction Enzyme	Allele (bp)
rs61330082	F: 5′-TGTTTCAAACCTCGTTGCTG-3′	*T/C*	203	62	ScrFI	C (65 + 138)
	R: 5′-GAGGCATGGCTGAGACTTCTA-3′					T (203)
rs2505568	F: 5′-AAGCTTTTTAGGGCCCTTTG-3′	*T/A*	233	62	RsaI	A (233)
	R: 5′-TCATGAAAAGTTGGAAAGACTGTT-3′					T (167 + 66)
rs9034	F: 5′-ATGTTTATTAACCTGCCCTTTACACAGAA-3′	*C/T*	131	62	HinfI	T (131)
	R: 5′-AATTATTTAGCCTCCTCCCTTCC-3′					C (99 + 32)

SNP, single nucleotide polymorphism; PCR, polymerase chain reaction.

### 4.5. Statistical Analysis

Data were analyzed using SPSS for Windows software package version 13.0 (SPSS Inc., Chicago, IL, USA). Genotype frequencies of these two SNPs were obtained by directed counting and Hardy-Weinberg equilibrium were evaluated by chi-square test. Odds ratio (OR) and respective 95% confidence intervals were reported to evaluate the effects of any difference between alleles, genotypes. Linkage disequilibrium (LD) among the three SNPs and haplotype analysis were used by SHESIS software. (Available online: http://analysis.bio-x.cn/myAnalysis.php). The *NAMPT* haplotypes of individuals were generated using the expectation maximization algorithm in PLINK software (Available online: http://pngu.mgh.harvard.edu/~purcell/plink/; Center for Human Genetic Research (CHGR), Massachusetts General Hospital (MGH), and the Broad Institute of Harvard & MIT, MA, USA). Power calculations were made by Power and Sample Size Calculation (Available online: http://biostat.mc.vanderbilt.edu/wiki/Main/PowerSampleSize). A univariate and multivariate Cox proportional-hazards regression analysis was performed to assess the association of genetic and clinical variables with the end point of cardiac death. Continuous variable of BNP was redefined as categorical according to median BNP plasma level. Univariate analysis was used by two-sided log-rank tests or Cox univariate analyses.

Statistically significant results in univariate analysis and other important clinical variables were further tested in multivariable models, including SNP rs9034 genotype, age, gender, creatinine, NYHA functional class, LVEF, BNP and β-blocker therapy. Continuous data were presented as means ± SD. Comparisons between groups for continuous data were made using the Kruskal-Wallis and Mann-Whitney nonparametric tests. Correlation between variables was determined using Spearman’s correlation test. A raw *p* value of <0.05 was considered nominally significant, which was further subjected to Bonferroni correction to account for multiple comparisons. The significance threshold was set at a *p* value of less than 0.017 for single SNP test (0.05/3 SNPs that were included in the association analyses) and 0.01 for haplotype analysis (0.05/5 haplotypes that were included in the association analyses).

## 5. Limitations

There are several potential limitations in this study. Due to China’s recent rapid urbanization, only 175 patients can be regularly followed up. The modest sample size of our followed up patients may have introduced a degree of bias. This is a single-center study. Studies with a larger sample size are needed to confirm our result and should be carried out in different populations worldwide. Finally, we did not look at the underlying mechanisms behind our findings.

## 6. Conclusions

In conclusion, our study demonstrates for the first time that rs9034 of the *NAMPT* gene was associated with susceptibility to DCM, and *TAC* (rs61330082-rs2505568-rs9034) was a protective haplotype to DCM. Rs9034 may be a novel genetic biomarker for prognosis of DCM. Rs2505568 also had a nominally significant association with DCM risk, but it should be interpreted with caution because of Hardy-Weinberg disequilibrium in the control group. Serum NAMPT levels were associated with the degree of heart failure and the LVEDD/LVEDV of echocardiography in DCM patients. Our study suggested NAMPT may play an important role in the development of DCM. Further studies are warranted to expand our results and to explore the mechanism of NAMPT in the pathogenesis of DCM.

## References

[1] Elliott P., Andersson B., Arbustini E., Bilinska Z., Cecchi F., Charron P., Dubourg O., Kuhl U., Maisch B., McKenna W.J. (2008). Classification of the cardiomyopathies: A position statement from the european society of cardiology working group on myocardial and pericardial diseases. Eur. Heart J..

[2] Sugrue D.D., Rodeheffer R.J., Codd M.B., Ballard D.J., Fuster V., Gersh B.J. (1992). The clinical course of idiopathic dilated cardiomyopathy. A population-based study. Ann. Intern. Med..

[3] Stehlik J., Edwards L.B., Kucheryavaya A.Y., Benden C., Christie J.D., Dipchand A.I., Dobbels F., Kirk R., Rahmel A.O., Hertz M.I. (2012). The registry of the international society for heart and lung transplantation: 29th official adult heart transplant report—2012. J. Heart Lung Transplant..

[4] Garcia-Pavia P., Cobo-Marcos M., Guzzo-Merello G., Gomez-Bueno M., Bornstein B., Lara-Pezzi E., Segovia J., Alonso-Pulpon L. (2013). Genetics in dilated cardiomyopathy. Biomark. Med..

[5] Ruppert V., Meyer T., Struwe C., Petersen J., Perrot A., Posch M.G., Ozcelik C., Richter A., Maisch B., Pankuweit S. (2010). Evidence for CTLA4 as a susceptibility gene for dilated cardiomyopathy. Eur. J. Hum. Genet..

[6] Chen Y., Zhou B., Peng Y., Wang Y., Li C., Ding X., He X., Xu J., Huang L., Rao L. (2009). Interleukin-23 receptor gene polymorphisms is associated with dilated cardiomyopathy in Chinese Han population. Tissue Antigens.

[7] Rongvaux A., Shea R.J., Mulks M.H., Gigot D., Urbain J., Leo O., Andris F. (2002). Pre-B-cell colony-enhancing factor, whose expression is up-regulated in activated lymphocytes, is a nicotinamide phosphoribosyltransferase, a cytosolic enzyme involved in NAD biosynthesis. Eur. J. Immunol..

[8] Kitani T., Okuno S., Fujisawa H. (2003). Growth phase-dependent changes in the subcellular localization of pre-B-cell colony-enhancing factor. FEBS Lett..

[9] Belenky P., Bogan K.L., Brenner C. (2007). NAD^+^ metabolism in health and disease. Trends Biochem. Sci..

[10] Tanno M., Kuno A., Horio Y., Miura T. (2012). Emerging beneficial roles of sirtuins in heart failure. Basic Res. Cardiol..

[11] Imai S. (2009). Nicotinamide phosphoribosyltransferase (Nampt): A link between NAD biology, metabolism, and diseases. Curr. Pharm. Des..

[12] Zhang L.Q., Heruth D.P., Ye S.Q. (2011). Nicotinamide phosphoribosyltransferase in human diseases. J. Bioanal. Biomed..

[13] Moschen A.R., Kaser A., Enrich B., Mosheimer B., Theurl M., Niederegger H., Tilg H. (2007). Visfatin, an adipocytokine with proinflammatory and immunomodulating properties. J. Immunol..

[14] Higuchi Y., Chan T.O., Brown M.A., Zhang J., DeGeorge B.R., Funakoshi H., Gibson G., McTiernan C.F., Kubota T., Jones W.K. (2006). Cardioprotection afforded by NF-κB ablation is associated with activation of Akt in mice overexpressing TNF-α. Am. J. Physiol. Heart Circ. Physiol..

[15] Wang X.H., Dou L.Z., Gu C., Wang X.Q. (2014). Plasma levels of omentin-1 and visfatin in senile patients with coronary heart disease and heart failure. Asian Pac. J. Trop. Med..

[16] Kozomara A., Griffiths-Jones S. (2011). miRBase: Integrating microRNA annotation and deep-sequencing data. Nucleic Acids Res..

[17] Zhang K., Zhou B., Zhang P., Zhang Z., Chen P., Pu Y., Song Y., Zhang L. (2014). Genetic variants in NAMPT predict bladder cancer risk and prognosis in individuals from southwest Chinese Han group. Tumour Biol..

[18] Wang B., Hasan M.K., Alvarado E., Yuan H., Wu H., Chen W.Y. (2011). NAMPT overexpression in prostate cancer and its contribution to tumor cell survival and stress response. Oncogene.

[19] Revollo J.R., Grimm A.A., Imai S. (2004). The NAD biosynthesis pathway mediated by nicotinamide phosphoribosyltransferase regulates Sir2 activity in mammalian cells. J. Biol. Chem..

[20] Alcendor R.R., Gao S., Zhai P., Zablocki D., Holle E., Yu X., Tian B., Wagner T., Vatner S.F., Sadoshima J. (2007). Sirt1 regulates aging and resistance to oxidative stress in the heart. Circ. Res..

[21] Kawashima T., Inuzuka Y., Okuda J., Kato T., Niizuma S., Tamaki Y., Iwanaga Y., Kawamoto A., Narazaki M., Matsuda T. (2011). Constitutive SIRT1 overexpression impairs mitochondria and reduces cardiac function in mice. J. Mol. Cell. Cardiol..

[22] Oka S., Alcendor R., Zhai P., Park J.Y., Shao D., Cho J., Yamamoto T., Tian B., Sadoshima J. (2011). PPARα-Sirt1 complex mediates cardiac hypertrophy and failure through suppression of the ERR transcriptional pathway. Cell Metab..

[23] Planavila A., Dominguez E., Navarro M., Vinciguerra M., Iglesias R., Giralt M., Lope-Piedrafita S., Ruberte J., Villarroya F. (2012). Dilated cardiomyopathy and mitochondrial dysfunction in Sirt1-deficient mice: A role for Sirt1-Mef2 in adult heart. J. Mol. Cell. Cardiol..

[24] Imai S. (2009). The NAD World: A new systemic regulatory network for metabolism and aging—Sirt1, systemic NAD biosynthesis, and their importance. Cell Biochem. Biophys..

[25] Zhang T., Berrocal J.G., Frizzell K.M., Gamble M.J., DuMond M.E., Krishnakumar R., Yang T., Sauve A.A., Kraus W.L. (2009). Enzymes in the NAD^+^ salvage pathway regulate SIRT1 activity at target gene promoters. J. Biol. Chem..

[26] Jia S.H., Li Y., Parodo J., Kapus A., Fan L., Rotstein O.D., Marshall J.C. (2004). Pre-B cell colony-enhancing factor inhibits neutrophil apoptosis in experimental inflammation and clinical sepsis. J. Clin. Investig..

[27] Ye S.Q., Simon B.A., Maloney J.P., Zambelli-Weiner A., Gao L., Grant A., Easley R.B., McVerry B.J., Tuder R.M., Standiford T. (2005). Pre-B-cell colony-enhancing factor as a potential novel biomarker in acute lung injury. Am. J. Respir Crit. Care Med..

[28] Brentano F., Schorr O., Ospelt C., Stanczyk J., Gay R. E., Gay S., Kyburz D. (2007). Pre-B cell colony-enhancing factor/visfatin, a new marker of inflammation in rheumatoid arthritis with proinflammatory and matrix-degrading activities. Arthritis Rheum..

[29] Tilg H., Moschen A.R. (2008). Role of adiponectin and PBEF/visfatin as regulators of inflammation: Involvement in obesity-associated diseases. Clin. Sci..

[30] Koczan D., Guthke R., Thiesen H. J., Ibrahim S.M., Kundt G., Krentz H., Gross G., Kunz M. (2005). Gene expression profiling of peripheral blood mononuclear leukocytes from psoriasis patients identifies new immune regulatory molecules. Eur. J. Dermatol..

[31] Dahl T.B., Yndestad A., Skjelland M., Oie E., Dahl A., Michelsen A., Damas J.K., Tunheim S.H., Ueland T., Smith C. (2007). Increased expression of visfatin in macrophages of human unstable carotid and coronary atherosclerosis: Possible role in inflammation and plaque destabilization. Circulation.

[32] Lu L.F., Wang C.P., Yu T.H., Hung W.C., Chiu C.A., Chung F.M., Tsai I.T., Yang C.Y., Cheng Y.A., Lee Y.J. (2012). Interpretation of elevated plasma visfatin concentrations in patients with ST-elevation myocardial infarction. Cytokine.

[33] Wang L.S., Yan J.J., Tang N.P., Zhu J., Wang Y.S., Wang Q.M., Tang J.J., Wang M.W., Jia E.Z., Yang Z.J. (2011). A polymorphism in the visfatin gene promoter is related to decreased plasma levels of inflammatory markers in patients with coronary artery disease. Mol. Biol. Rep..

[34] Bobbert P., Kuhl U., Poller W., Rauch U., Schultheiss H.P., Skurk C. (2015). Nicotinamide phosphoribosyltransferase/pre-B-cell colony enhancing factor/visfatin plasma levels and clinical outcome in patients with dilated cardiomyopathy. J. Card. Fail..

[35] O’Mahony D.S., Glavan B.J., Holden T.D., Fong C., Black R.A., Rona G., Tejera P., Christiani D.C., Wurfel M.M. (2012). Inflammation and immune-related candidate gene associations with acute lung injury susceptibility and severity: A validation study. PLoS ONE.

[36] Leander K., Gigante B., Silveira A., Vikstrom M., Hamsten A., Hogberg J. (2012). NAMPT (visfatin) and AKT1 genetic variants associate with myocardial infarction. Clin. Chim. Acta.

[37] Garcia-Bermudez M., Gonzalez-Juanatey C., Rodriguez-Rodriguez L., Miranda-Filloy J.A., Perez-Esteban S., Vazquez-Rodriguez T.R., Castaneda S., Balsa A., Fernandez-Gutierrez B., Llorca J. (2011). Lack of association of NAMPT rs9770242 and rs59744560 polymorphisms with disease susceptibility and cardiovascular risk in patients with rheumatoid arthritis. Clin. Exp. Rheumatol..

[38] Blank M.C., Stefanescu R.N., Masuda E., Marti F., King P.D., Redecha P.B., Wurzburger R.J., Peterson M.G., Tanaka S., Pricop L. (2005). Decreased transcription of the human *FCGR2B* gene mediated by the −343 G/C promoter polymorphism and association with systemic lupus erythematosus. Hum. Genet..

[39] Kim B.C., Kim W.Y., Park D., Chung W.H., Shin K.S., Bhak J. (2008). SNP@Promoter: A database of human SNPs (single nucleotide polymorphisms) within the putative promoter regions. BMC Bioinform..

[40] Bartel D.P. (2004). MicroRNAs: Genomics, biogenesis, mechanism, and function. Cell.

[41] Sethupathy P., Borel C., Gagnebin M., Grant G.R., Deutsch S., Elton T.S., Hatzigeorgiou A.G., Antonarakis S.E. (2007). Human microRNA-155 on chromosome 21 differentially interacts with its polymorphic target in the AGTR1 3′ untranslated region: A mechanism for functional single-nucleotide polymorphisms related to phenotypes. Am. J. Hum. Genet..

[42] Chin L.J., Ratner E., Leng S., Zhai R., Nallur S., Babar I., Muller R.U., Straka E., Su L., Burki E.A. (2008). A SNP in a let-7 microRNA complementary site in the KRAS 3′ untranslated region increases non-small cell lung cancer risk. Cancer Res..

[43] Trikalinos T.A., Salanti G., Khoury M.J., Ioannidis J.P. (2006). Impact of violations and deviations in Hardy-Weinberg equilibrium on postulated gene-disease associations. Am. J. Epidemiol..

[44] Moschen A.R., Gerner R.R., Tilg H. (2010). Pre-B cell colony enhancing factor/NAMPT/visfatin in inflammation and obesity-related disorders. Curr. Pharm. Des..

[45] Richardson P., McKenna W., Bristow M., Maisch B., Mautner B., O’Connell J., Olsen E., Thiene G., Goodwin J., Gyarfas I. (1996). Report of the 1995 World Health Organization/International Society and Federation of Cardiology Task Force on the Definition and Classification of cardiomyopathies. Circulation.

[46] Maron B.J., Towbin J.A., Thiene G., Antzelevitch C., Corrado D., Arnett D., Moss A.J., Seidman C.E., Young J.B. (2006). Contemporary definitions and classification of the cardiomyopathies an American heart association scientific statement from the council on clinical cardiology, heart failure and transplantation committee; quality of care and outcomes research and functional genomics and translational biology interdisciplinary working groups; and council on epidemiology and prevention. Circulation.

[47] Schiller N.B., Shah P.M., Crawford M., DeMaria A., Devereux R., Feigenbaum H., Gutgesell H., Reichek N., Sahn D., Schnittger I. (1989). Recommendations for quantitation of the left ventricle by two-dimensional echocardiography. American Society of Echocardiography Committee on Standards, Subcommittee on Quantitation of Two-Dimensional Echocardiograms. J. Am. Soc. Echocardiogr..

